# Optical Coherence Tomography Findings in Patients Presenting With In-Stent Restenosis: A Prospective Observational Study of Patterns of Neointimal Hyperplasia and Associated Risk Factors

**DOI:** 10.7759/cureus.46888

**Published:** 2023-10-12

**Authors:** Gaurav Chaudhary, Javed Akhtar, Shubhajeet Roy, Timil Suresh, Jay Tewari, Ayush Shukla, Sharad Chandra, Akhil Sharma, Akshyaya Pradhan, Monika Bhandari, Pravesh Vishwakarma, Rishi Sethi, Abhishek Singh, Sudhanshu K Dwivedi

**Affiliations:** 1 Cardiology, King George's Medical University, Lucknow, IND; 2 Faculty of Medicine, King George's Medical University, Lucknow, IND; 3 Internal Medicine, King George's Medical University, Lucknow, IND

**Keywords:** percutaneous coronary intervention, optical coherence tomography, neointimal hyperplasia, neoatherosclerosis, intracoronary imaging, in-stent restenosis, drug-eluting stent, coronary artery disease

## Abstract

Introduction

Morphological features of neointimal tissue play a pivotal role in the pathophysiology of in-stent restenosis (ISR) after percutaneous coronary intervention (PCI). This study was designed to qualitatively and quantitatively assess neointimal characteristics of lesions using optical coherence tomography (OCT) in patients presenting with ISR.

Methods

This was a single-center, prospective, observational study performed at a tertiary-care center in India. Patients diagnosed with stable angina and acute coronary syndrome with post-procedural angiographically documented restenosis (>50%) were included.

Results

A total of 34 patients with ISR were studied. Neointimal hyperplasia was classified as (i) homogenous group (n = 18) and (ii) non-homogenous group (n = 16). Fourteen (77.8%) diabetics belonged to the homogenous group. Predominant plaque characteristics such as neoatherosclerosis, cholesterol crystals, and calcium were documented in 14 (77.8%), 12 (66.7%), and 11 (61.1%) patients in the homogenous group and 10 (62.5%), 10 (62.5%), and 9 (56.2%) patients in the non-homogenous group, respectively. Unexpanded stent struts were identified in 11 (61.1%) and 11 (68.8%) patients in the homogenous and non-homogenous groups, respectively. Mean strut thickness was 93.73 ± 31.03 µm and 83.54 ± 18.0 µm, ISR was 72.50 ± 15.93% and 65.37 ± 21.69%, the neointimal thickness was 588.06 ± 167.82 μm and 666.25 ± 218.05 μm, and neointimal hyperplasia was 54.54 ± 11.23% and 59.26 ± 8.86% in the homogenous and non-homogenous groups, respectively.

Conclusion

Neoatherosclerosis and stent underexpansion were predominantly observed in our study and only diabetes was found to be significantly associated with homogenous neointimal hyperplasia.

## Introduction

In-stent restenosis (ISR) is triggered by suboptimal re-endothelization and exaggerated neointimal proliferation which can occur within months or years after stent implantation [1]. The bare-metal stent era witnessed a 32-55% incidence of ISR whereas the introduction of second-generation drug-eluting stents prompted a decline in ISR incidence to 10-12.2% [2-4]. Nonetheless, ISR still prevails to a lesser extent in routine clinical practice [5]. Intracoronary optical coherence tomography (OCT) provides a higher axial resolution than conventional intravascular ultrasound. In a recently conducted multicentric randomized controlled trial by Ali ZA, et al. in 2023, it was observed that the minimum stent area after percutaneous coronary intervention (PCI) was 5.72 ± 2.04 mm^2^ in the OCT group and 5.36 ± 1.87 mm^2^ in the angiography group (p < 0.001). Target-vessel failure within two years occurred in 88 patients in the OCT group and in 99 patients in the angiography group (p = 0.45), OCT-related adverse events occurred in one patient in the OCT group and in two patients in the angiography group, and stent thrombosis within two years occurred in six patients (0.5%) in the OCT group and in 17 patients (1.4%) in the angiography group [6]. It also proves useful for qualitative as well as quantitative evaluation of neointimal tissue [7]. In earlier studies, it was hypothesized that the morphological characteristics underlying the ISR lesion, that is neoatherosclerotic or peri-stent calcium, would have an impact on the expansion of an implanted stent during revascularization. These morphologic characteristics can be evaluated using OCT providing highlights of the tissue covering the stent struts at follow-up [8]. Pathological findings revealed that neointima within a stent consists of numerous tissue components including collagen, proteoglycan, smooth muscle, fibrin, and thrombus. This observation is suggestive of differential prognosis of stented lesions according to in-stent neointimal characteristics [9]. Although previous studies have explored correlations between neointimal patterns, underlying stent type, and restenosis phase, the degree of intralesional neointimal characteristics has not been elucidated [5,10,11]. Therefore, this study sought to qualitatively and quantitatively assess neointimal characteristics of lesions using OCT in patients presenting with ISR. Studying the patterns of ISR is very important and has clinical implications in planning the strategy of PCI after OCT imaging. For example, if the type of ISR is neoatherosclerosis with calcium (calcific neoatherosclerosis), cutting balloon/scoring balloon can be employed as the preferred modality of PCI, whereas if the type of ISR is severely calcified neoatherosclerosis, rotablation or intravascular lithotripsy can be employed as the preferred modality of PCI.

This article was previously presented as a poster at the ESC (European Society of Cardiology) ASIA 2022 with the Asian Pacific Society of Cardiology (APSC) and Asean Federation of Cardiology (AFC), at Raffles City Convention Centre, Singapore.

## Materials and methods

Study design and patient population

This was a single-center, prospective observational study conducted at a tertiary care center in India between 1 August 2020 and 30 December 2021. A total of 34 consecutive post-PCI patients, diagnosed with stable angina and acute coronary syndrome, who had undergone invasive coronary angiography followed by OCT, were enrolled. The exclusion criteria were patients with (a) known systolic heart failure with left-ventricular ejection fraction ≤30%; (b) cardiogenic shock or heart failure requiring intubation, inotropes, diuretics, or mechanical circulation support; (c) refractory ventricular arrhythmia requiring pharmacologic or defibrillator therapy; (d) renal insufficiency with serum creatinine ≥1.5 mg/dL; (e) diagnosis of acute coronary syndrome with culprit lesion in a bypass graft; and (f) unsuitable "culprit lesion" defined by OCT as severe calcification or extreme tortuosity of the culprit lesion, culprit lesion with distal location, or infarct vessels with diameter >4 mm or <2 mm. The study was performed in accordance with the Declaration of Helsinki, and the study protocol was approved by the Institutional Ethics Committee. All patients provided written informed consent prior to participation in the study.

OCT image acquisition

OCT images were acquired using a frequency-domain OCT system (C7-XR OCT Intravascular Imaging System; St. Jude Medical Inc., St. Paul, MN). OCT images were generated at 100 frames/s and the catheter was pulled back at a speed of 20 mm/s. A contrast medium was continuously flushed through a guiding catheter at a rate of 4-5 mL/s for 3-4 s. Continuous images were acquired and stored digitally for subsequent analysis [7].

OCT image analysis

The OCT examination was performed using off-line OCT (Light Lab Imaging Inc., Westford, MA) software. For qualitative analysis, the pattern of re-stenosed tissue structure in the cross-sectional images at every 1 mm interval was categorized into two types: homogeneous and non-homogenous patterns of neointimal hyperplasia (NIH), of which, non-homogenous was further divided into heterogenous and layered patterns. 

Quantitative analysis of OCT images was performed at minimal lumen area (MLA) sites. The MLA and stent area were manually traced, and the mean neointimal thickness was automatically calculated. Stent and luminal cross-sectional area (CSA) were measured, and NIH CSA was then calculated as the stent CSA minus the luminal CSA. The percentage of NIH-CSA was calculated as NIH-CSA × 100/stent CSA. The NIH thickness, the distance between the endoluminal surface of the neointima and the strut, can be measured inside all the struts as a line perpendicular to the neointima and the strut. 

Cross-sectional OCT images of in-stent segments were analyzed at every 1 mm of the stent body. The region of interest was defined as an in-stent segment and 5 mm proximal and 5 mm distal segments. An uncovered strut was defined as having an NIH thickness of 0 µm. Malapposition was defined as the separation of stent struts from the vessel wall with a strut-vessel lumen distance of >200 mm.

Definitions

**Figure 1:**
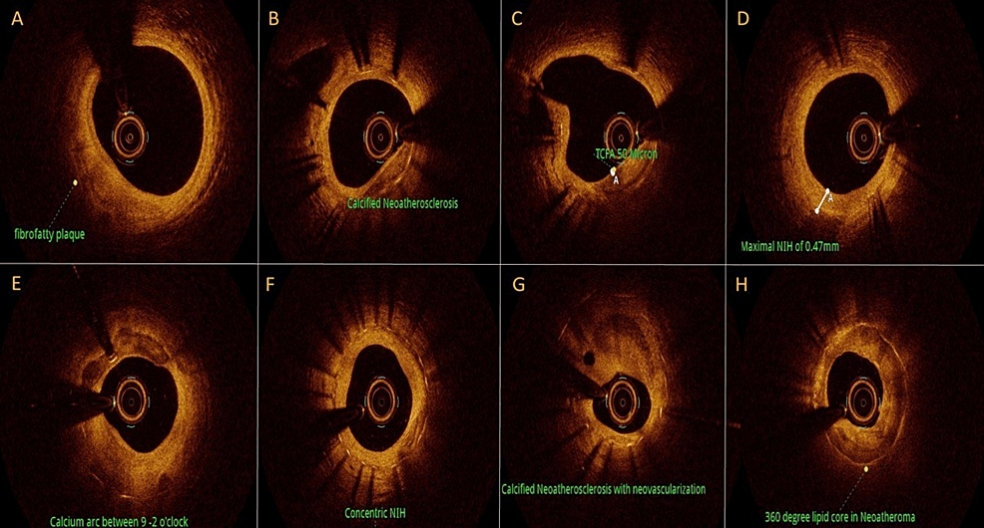
OCT findings: (A) fibrofatty plaque; (B) calcified neoatherosclerosis; (C) thin-cap fibroatheroma; (D) neointimal hyperplasia; (E) calcium arcs; (F) concentric neointimal hyperplasia; (G) calcified neoatherosclerosis with neovascularization; and (H) 360 degree lipid core in neoatheroma. TCFA: thin-cap fibroatheroma; NIH: neointimal hyperplasia; OCT: optical coherence tomography.

ISR was defined as in-stent segments - 5 mm proximal and distal segments. Briefly, homogeneous tissues have uniform optical properties without focal variation in the backscattering pattern, heterogeneous tissues have focally changing optical properties and various backscattering patterns, and layered tissues consist of concentric layers with different optical properties [9]. Thrombus was defined as signal-rich, low-backscattering protrusions (white thrombus), or high-backscattering protrusions (red thrombus) inside the lumen with signal-free shadowing. Neoatherosclerosis was defined as the presence of lipid-laden intima and/or calcification inside the stent. Thin-cap fibroatheroma-like neointima was defined as the presence of an area with signal attenuation and a diffuse border, along with <65 µm fibrous cap thickness at the thinnest part. The site of neoatherosclerosis was classified as the proximal section, the middle section, and the distal section of the stent body. Micro-vessel was defined as a small tubular or vesicular structure with a diameter of <200 µm [12] (Figure 1).

Statistical analysis

The Statistical Package for Social Sciences (SPSS; IBM Corp, Armonk, NY) program, version 21.0, was used for data analysis. Continuous variables were expressed as mean ± standard deviation and categorical variables as percentages. Data normality was checked using the Kolmogorov-Smirnov test. Independent t-test and Mann-Whitney test were used for normally and non-normally distributed data, respectively. Categorical variables were compared with the chi-square test or Fisher’s exact test. A p-value of <0.05 was considered statistically significant.

## Results

Baseline demographic characteristics

A total of 34 patients with ISR were studied, in whom quantitative and qualitative plaque characteristics were analyzed using OCT. The mean age of the patients was 54.79 ± 9.98 years. The majority of the patients belonged to the 51-65 years age category. Homogenous NIH was observed in 18 (52.9%) patients while non-homogenous NIH was observed in 16 (47.1%) patients. Of the 16 patients with non-homogenous NIH, 14 (87.5%) patients had a heterogenous pattern and 2 (12.5%) patients had a layered pattern of NIH. There was male predominance, i.e., 13 (72.2%) and 13 (81.2%) males in the homogenous and non-homogenous groups, respectively. The mean prior PCI interval for the homogenous group was 6.61 ± 4.94 years and for the non-homogenous group it was 4.88 ± 3.83 years. Diabetes mellitus was found in 14 (77.8%) and 5 (31.2%) patients (p = 0.014) among the homogenous and non-homogenous groups, respectively. History of coronary artery disease was documented in 12 (66.7%) and 12 (75%) patients (p = 0.595) in the homogenous and non-homogenous groups, respectively. In this study, the majorly affected vessel with ISR was the left anterior descending artery in 10 (55.6%) and 9 (56.2%) among the homogenous and non-homogenous groups, respectively. Baseline and angiographic characteristics among the homogeneous and non-homogeneous groups are demonstrated in Table 1. 

**Table 1:** Baseline and angiographic characteristics among homogenous and non-homogenous groups All data are expressed as number (percentage). H/O CAD: history of coronary artery disease; ACS: acute coronary syndrome; NSTEMI: non-ST-elevated myocardial infarction; AWMI: anterior wall myocardial infarction; IWMI: inferior wall myocardial infarction; CAD: coronary artery disease; CSA: chronic stable angina; USA: unstable angina.

	Homogenous (n = 18)	Non-homogenous (n = 16)	Total (n = 34)	p-Value
Age intervals				
35-50 years	6 (33.3%)	6 (37.5%)	12 (35.3%)	0.411
51-65 years	11 (61.1%)	7 (43.8%)	18 (52.9%)	
>65 years	1 (5.6%)	3 (18.8%)	4 (11.8%)	
Gender				
Male	13 (72.2%)	13 (81.2%)	26 (76.5%)	0.693
Female	5 (27.8%)	3 (18.8%)	8 (23.5%)	
Risk factors				
H/O CAD	12 (66.7%)	12 (75%)	24 (70.6%)	0.595
Diabetes mellitus	14 (77.8%)	5 (31.2%)	19 (55.9%)	0.014
Family history	8 (44.4%)	11 (68.8%)	19 (55.9%)	0.154
Hypertension	7 (38.9%)	7 (43.8%)	14 (41.2%)	0.774
Smoker	5 (27.8%)	7 (43.8%)	12 (35.3%)	0.331
Tobacco chewer	2 (11.1%)	5 (31.2%)	7 (20.6%)	0.214
Drug defaulter	0 (0%)	0 (0%)	0 (0%)	NA
Prior diagnosis				
ACS NSTEMI	7 (38.9%)	5 (31.2%)	12 (35.3%)	0.922
AWMI/ IWMI	7 (38.9%)	8 (50%)	15 (44.1%)	
CAD/CSA	3 (16.7%)	2 (12.5%)	5 (14.7%)	
USA	1 (5.6%)	1 (6.2%)	2 (5.9%)	
Present diagnosis				
ACS NSTEMI	3 (16.7%)	3 (18.8%)	6 (17.6%)	0.944
AWMI/IWMI	3 (16.7%)	3 (18.8%)	6 (17.6%)	
CAD/CSA	7 (38.9%)	7 (43.8%)	14 (41.2%)	
USA	5 (27.8%)	3 (18.8%)	8 (23.5%)	
Vessel with ISR				
LAD	10 (55.6%)	9 (56.2%)	19 (55.9%)	0.699
LCX	4 (22.2%)	2 (12.5%)	6 (17.6%)	
RCA	4 (22.2%)	5 (31.2%)	9 (26.5%)	

Optical coherence tomographic analysis 

Thrombolysis was performed in 3 (16.7%) and 3 (18.8%) patients (p = 1.000) among the homogenous and non-homogenous groups, respectively. Plaque characteristics such as neoatherosclerosis, cholesterol crystal, and calcium were observed in 14 (77.8%) and 10 (62.5%) patients (p = 0.329), 12 (66.7%) and 10 (62.5%) patients (p = 0.800), and 11 (61.1%) and 9 (56.2%) patients (p = 0.774), among the homogenous and non-homogenous groups, respectively. Stent under expansion was observed in 11 (61.1%) and 11 (68.8%) patients (p = 0.642) among the homogenous and non-homogenous groups, respectively. Malapposed struts were found in 4 (22.2%) and 6 (37.5%) patients (p = 0.329), among the homogenous and non-homogenous groups, respectively. Diffuse restenosis pattern was detected in 11 (61.1%) and 11 (68.8%) patients among the homogenous and non-homogenous groups, respectively. Plaque and stent characteristics among the homogenous and non-homogenous groups are detailed in Table 2.

**Table 2:** Plaque and stent characteristics among homogenous and non-homogenous groups All data are expressed as number (percentage).

Variable	Homogenous (n = 18)	Non-homogenous (n = 16)	Total (n = 34)	p-Value
Thrombolysis	3 (16.7%)	3 (18.8%)	6 (17.6%)	1.000
Plaque characteristic				
Neoatherosclerosis	14 (77.8%)	10 (62.5%)	24 (70.6%)	0.329
Micro-vessels	9 (50%)	8 (50%)	17 (50%)	1.000
Cholesterol crystal	12 (66.7%)	10 (62.5%)	22 (64.7%)	0.800
Macrophage	9 (50%)	7 (43.8%)	16 (47.1%)	0.716
Calcium	11 (61.1%)	9 (56.2%)	20 (58.8%)	0.774
Thrombus	6 (33.3%)	6 (37.5%)	12 (35.3%)	0.800
Stent characteristic				
Uncovered strut	5 (27.8%)	3 (18.8%)	8 (23.5%)	0.693
Malapposed strut	4 (22.2%)	6 (37.5%)	10 (29.4%)	0.329
Stent underexpansion	11 (61.1%)	11 (68.8%)	22 (64.7%)	0.642
Pattern of restenosis				
Diffuse	11 (61.1%)	11 (68.8%)	22 (64.7%)	0.748
Focal	4 (22.2%)	4 (25%)	8 (23.5%)	
Multifocal	1 (5.6%)	00	1 (2.9%)	
Proliferative	2 (11.1%)	1 (6.2%)	3 (8.8%)	

Laboratory findings

Lipid profiles such as total cholesterol (141.07 ± 30.10 mg/dL and 140.81 ± 39.82 mg/dL, p = 0.983), triglyceride (169.31 ± 68.15 mg/dL and 170.46 ± 130.40 mg/dL, p = 0.974), high-density lipoprotein (42.59 ± 10.17 mg/dL and 45.52 ± 11.11 mg/dL, p = 0.428), and low-density lipoprotein (82.56 ± 34.22 mg/dL and 79.71 ± 38.33 mg/dL, p = 0.820) were reported among the homogenous versus non-homogenous group, respectively. Laboratory findings among the homogenous and non-homogenous groups are represented in Table 3. 

**Table 3:** Comparison of laboratory findings among homogenous and non-homogenous groups All data are expressed as mean ± standard deviation. HDL: high-density lipoprotein; LDL: low-density lipoprotein; PCI: percutaneous coronary intervention.

Variable	Homogenous (n = 18)	Non-homogenous (n = 16)	Total (n = 34)	p-Value
Total cholesterol, mg/dL	141.07 ± 30.10	140.81 ± 39.82	140.94 ± 34.46	0.983
Triglyceride, mg/dL	169.31 ± 68.15	170.46 ± 130.40	169.85 ± 100.61	0.974
HDL, mg/dL	42.59 ± 10.17	45.52 ± 11.11	43.97 ± 10.56	0.428
LDL, mg/dL	82.56 ± 34.22	79.71 ± 38.33	81.22 ± 35.68	0.820
Ejection fraction, %	52.33 ± 7.87	51.87 ± 10.39	52.12 ± 9.00	0.885
Serum creatinine, mg/dL	1.08 ± 0.32	1.03 ± 0.26	1.06 ± 0.29	0.609

OCT-derived quantitative assessments of plaque characteristics

Strut thickness was documented as 93.73 ± 31.03 µm and 83.54 ± 18.06 µm among the homogenous and non-homogenous groups, respectively. Restenosis in vessels with ISR was documented as 72.50 ± 15.93% vs 65.37 ± 21.69% (p = 0.279) among the homogenous and non-homogenous groups, respectively. The length of the ISR lesion was noted as 24.62 ± 4.89 mm and 24.63 ± 7.02 mm (p = 0.994) and neointimal thickness was reported to be 588.06 ± 167.82 μm and 666.2 ± 218.05 μm (p = 0.247) among the homogenous and non-homogenous groups, respectively. Mean MLA was 2.22 ± 1.00 mm^2^ and 2.28 ± 0.95 mm^2^ (p = 0.856) among the homogenous and non-homogenous groups, respectively. Mean minimal lumen diameter (MLD) was 1.38 ± 0.33 mm and 1.36 ± 0.42 mm (p = 0.860) among the homogenous and non-homogenous groups, respectively. NIH was 54.54 ± 11.23% and 59.26 ± 8.86% (p = 0.187) among the homogenous and non-homogenous groups, respectively. OCT-derived quantitative assessments of plaque characteristics among the homogenous and non-homogenous groups are represented in Table 4.

**Table 4:** Optical coherence tomography-derived quantitative assessments of plaque characteristics among homogenous and non-homogenous groups All data are expressed as mean ± standard deviation. ISR: in-stent restenosis; TCFA: thin-cap fibroatheroma; MLA: minimal lumen area; MLD: minimal lumen diameter; NIH: neointimal hyperplasia.

Variable	Homogenous (n = 18)	Non-homogenous (n = 16)	Total (n = 34)	p-Value
Strut thickness, µm	93.73 ± 31.03	83.54 ± 18.06	89.00 ± 25.91	0.308
Restenosis in vessel with ISR, %	72.50 ± 15.93	65.37 ± 21.69	69.15 ± 18.91	0.279
Length of ISR lesion, mm	24.62 ± 4.89	24.63 ± 7.02	24.62 ± 5.89	0.994
Neointimal thickness, μm	588.06 ± 167.82	666.25 ± 218.05	624.85 ± 194.14	0.247
TCFA, µm	98.28 ± 21.13	100.88 ± 19.09	624.85 ± 194.14	0.711
MLA, mm^2^	2.22 ± 1.00	2.28 ± 0.95	2.25 ± 0.96	0.856
MLD, mm	1.38 ± 0.33	1.36 ± 0.42	1.37 ± 0.37	0.860
NIH, %	54.54 ± 11.23	59.26 ± 8.86	56.76 ± 10.31	0.187

## Discussion

We found the prevalence of homogenous NIH in 18 (52.9%) patients and non-homogenous in 16 (47.1%) patients. A similar prevalence was observed in a study by Kim et al. [9] who reported 207 (54.9%) patients with homogenous and 170 (45.1%) patients with non-homogenous NIH. A study by Xhepa et al. [10] demonstrated a significant difference between the homogenous and non-homogenous groups in diabetes mellitus (p = 0.026), although hypercholesterolemia has been an established risk factor for atherosclerosis [13]. The lipid profile of patients in the present study showed that there was no significant difference between the homogenous and non-homogenous groups. Similarly, in our study, diabetes mellitus was more common in the homogenous group, 14 (77.8%), compared to the non-homogenous group, 5 (31.2%). Neoatheroslecrosis progression occurs due to proteoglycan deposition which promotes the retention of lipoprotein and infiltration of inflammatory cells such as lymphocytes, macrophages, and giant cells. These cells may gradually replace smooth muscle cells, resulting in changes in the neointimal pattern [14]. ISR if seen at the histological level can be attributed to the accumulation of lipid-laden foamy macrophages with the presence or absence of a necrotic core, or calcification within the nascent intima after stent placement. The most common and the earliest lesion is foamy macrophage clusters, seen mostly in the luminal surface or in the peri-strut region. The above induces fibroatheroma formation. The core of necrosis mostly contains cell-free fragments with free cholesterol, with a mostly damaged extracellular matrix. Significant hemorrhage with fibrin accumulation could be seen in the necrotic core in neoatherosclerosis, originating after a fissure or rupture of the luminal surface or from leaky vasa vasorum developed into the adventitial layer near the stent struts. Also, foamy macrophages migrated into the neointimal layer can initiate the development of fibroatheroma with a thin cap, resulting in in-stent plaque rupture and thrombosis [15]. In the present study, plaque characteristics such as neoatherosclerosis among the homogenous and non-homogenous groups were reported in 14 (77.8%) and 10 (62.5%) patients, p = 0.329, respectively, which can be attributed to the mean prior PCI interval which is 5.79 years. The patient cohort in the present study is representative of patients vulnerable to the occurrence of late ISR. Our finding was in line with the previous study of Lee et al. [3] who identified 3 (14.3%) patients with early ISR and 36 (35.6%) patients with late ISR. In contrast to our study, Yamamoto et al. [14] reported progression to neoatherosclerosis in 0.6% of patients with a homogeneous pattern, 5.6% of patients with a heterogeneous pattern, and 3.9% of patients with a layered pattern at the six-month follow-up. Microvessels among the homogenous and non-homogenous groups were reported in 9 (50%) and 8 (50%) patients (p = 1.000), respectively. In contrast, a previous study reported the presence of microvessels in 22 (24.2%) and 6 (25.0%) patients among homogenous and heterogenous groups, respectively [16]. This difference in the prevalence of microvessels is due to the follow-up duration which was one year in the study of Shi et al. [16]. Pathologically, infiltration and accumulation of macrophages are supposed to be a vital process in the development of susceptible plaques [16]. In our study, macrophages were reported in 9 (50%) and 7 (43.8%) patients among the homogenous and non-homogenous groups, respectively. A previous study reported that mean macrophage detected by OCT is an independent morphological risk factor of neointimal heterogeneity [16]. Calcium among the homogenous and non-homogenous groups was reported in 11 (61.1%) and 9 (56.2%) patients (p = 0.774), respectively. However, Shi et al. [16] found calcified plaque in 39 (42.9%) and 7 (29.2%) among the homogenous and heterogenous groups, respectively.

In the present study, thrombus among the homogenous and non-homogenous groups was reported in 6 (33.3%) and 6 (37.5%) patients (p = 0.800), respectively. On the contrary, Shi et al. [16] in their study reported thrombus in 13 (14.3%) and 6 (25.0%) among the homogenous and heterogenous groups, respectively.

Stent underexpansion has long been recognized as a major mechanical factor triggering ISR. In the present study, stent underexpansion was found in 11 (61.1%) and 11 (68.8%) among the homogenous and non-homogenous groups, respectively. Similarly, a previous study by Xhepa et al. [10] reported stent underexpansion in 44 (61.1%) and 16 (48.5%) among the homogenous and non-homogenous groups, respectively. The uncovered strut in our study was reported in 5 (27.8%) and 3 (18.8%) among the homogenous and non-homogenous groups, respectively. Malapposed strut was reported in 4 (22.2%) and 6 (37.5%) among the homogenous and non-homogenous groups, respectively.

Quantitative assessments determined by OCT for homogenous and non-homogenous groups were strut thickness of 93.73 ± 31.03 µm and 83.54 ± 18.06 µm, respectively. In a study by Kitabata et al. [17], authors reported the possibility of thicker struts evoking a more pronounced inflammatory reaction to the metal. Neointimal thickness was 588.06 ± 167.82 μm and 666.25 ± 218.05 μm, and thin-cap fibroatheroma was 98.28 ± 21.13 µm and 100.88 ± 19.09 µm among the homogenous and non-homogenous groups, respectively. MLA was 2.22 ± 1.00 mm^2^ and 2.28 ± 0.95 mm^2^. These findings are in line with the previous studies of Kim et al. [9] where minimal lumen CSA was 4.5 ± 1.6 mm^2^ and 4.0 ± 1.9 mm^2^. MLD was reported as 1.38 ± 0.33 mm and 1.36 ± 0.42 mm, which coincides with the previous study of Jung et al. [11] where the mean lumen diameter was 1.1 ± 0.4 mm and 1.2 ± 0.4 mm. NIH in our study was 54.54 ± 11.23 and 59.26 ± 8.86. On the other hand, the previous findings of Habara et al. [18] reported NIH as 58.6 ± 10.3% and 55.6 ± 13.4% for very late ISR and early ISR. The length of the ISR lesions was 24.62 ± 4.89 mm and 24.63 ± 7.02 mm among the homogenous and non-homogenous groups, respectively. Chang et al. [19] observed that in the bare-metal stent group, the longer the lesion length, the higher the ISR rates. Similarly, stent length and lesion length have been reported as independent predictors of ISR in various drug-eluting stents such as sirolimus-eluting stents. However, there was no significant difference in the length of ISR lesions between the homogenous and non-homogenous groups in our study. Our study may have some limitations due to it being single-centric, having a relatively smaller sample size, and there may be interobserver variations in the OCT findings. Hence, a larger multicentric study is required in the future for more generalization of the results in real-world practice.

## Conclusions

The present study demonstrates the differential patterns of ISR tissue utilizing OCT. Predominantly, neoatherosclerosis and stent underexpansion were found to be associated with ISR. Out of various risk factors, only diabetes mellitus was found to be significantly associated with homogenous NIH. This classification and OCT findings could be advantageous for the interpretation and management of ISR.
